# Supplementary material for: Causal Relationship between Aging and Anorexia Nervosa: A White-Matter-Microstructure-Mediated Mendelian Randomization Analysis
Source: Biomedicines. 2024 Aug 16;12(8):1874. doi: 10.3390/biomedicines12081874 (PMC11351342; doi:10.3390/biomedicines12081874)
Supplement: Supplementary file 1 [file biomedicines-12-01874-s001.zip › Supplementary Figure/Figure S7/Single SNP plot of MR analysis for ubm-b-1920 to AN.pdf]

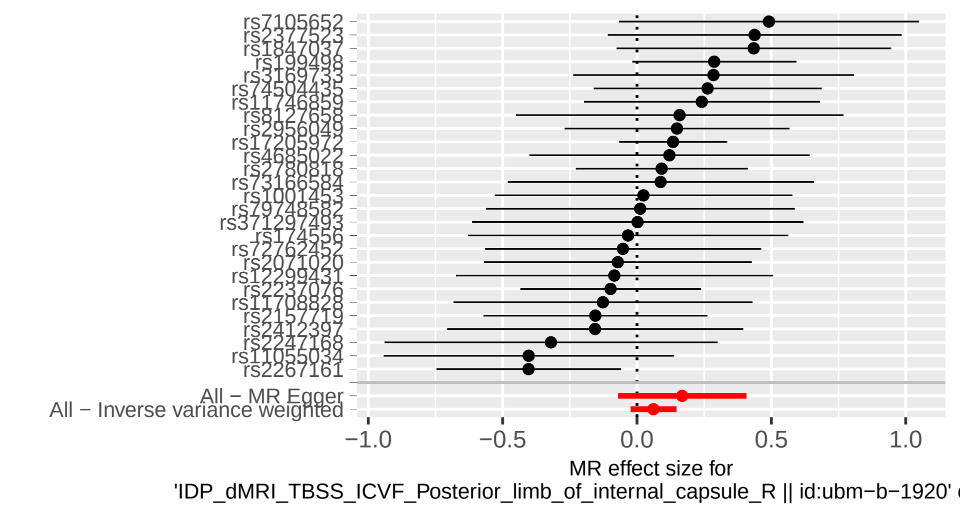
rs7105652
rs2377523
rs1847037
rs199498
rs3169733
rs74504435
rs11746859
rs8127658
rs2956049
rs17205972
rs4685022
rs2780818
rs73166584
rs1001453
rs79748582
rs371297493
rs174556
rs72762452
rs2071020
rs12299431
rs2237076
rs11708828
rs2157719
rs2412397
rs2247168
rs11055034
rs2267161
All − MR Egger
All − Inverse variance weighted
−1.0
−0.5
0.0
0.5
1.0
MR effect size for
'IDP_dMRI_TBSS_ICVF_Posterior_limb_of_internal_capsule_R || id:ubm−b−1920' o
